# Epigallocatechin-3-Gallate Promotes the Growth of Mink Hair Follicles Through Sonic Hedgehog and Protein Kinase B Signaling Pathways

**DOI:** 10.3389/fphar.2018.00674

**Published:** 2018-06-26

**Authors:** Haihua Zhang, Weixiao Nan, Shiyong Wang, Xingchao Song, Huazhe Si, Tong Li, Guangyu Li

**Affiliations:** ^1^State Key Laboratory of Special Economic Animal Molecular Biology, Institute of Special Animal and Plant Sciences, Chinese Academy of Agricultural Sciences, Changchun, China; ^2^College of Animal Science and Technology, Hebei Normal University of Science and Technology, Qinhuangdao, China; ^3^College of Animal Science and Technology, Jilin Agricultural University, Changchun, China; ^4^High-Tech Zone Laboratory of Public Test and Analysis Service, Shenyang, China

**Keywords:** dermal papilla cells, epigallocatechin-3-gallate, hair follicle growth, outer root sheath cells, protein kinase B, signaling pathway, sonic hedgehog signaling pathway

## Abstract

**Background:** Hair follicles play an essential role in the growth of hair. Epigallocatechin-3-gallate (EGCG), a catechin polyphenol in green tea, has various bioactivities. The present study aims to evaluate the effect of EGCG on the growth of mink hair follicles and investigate the possible molecular mechanisms.

**Methods:** The length of hair follicles was recorded up to 6 days in presence of 0.1–5 μM EGCG. Primary dermal papilla cells (DPCs) and outer root sheath cells (ORSCs) were treated with 0.25–4 μM EGCG, and their growth was evaluated by MTT assay and cell cycle detection. The levels of key molecules in sonic hedgehog (Shh) and protein kinase B (AKT) signaling pathways were further assessed by quantitative real-time PCR, western blot and immunofluorescence. To determine the involvement of Shh and AKT pathways in EGCG-mediated growth-promotion of ORSCs and DPCs, Shh pathway inhibitors cyclopamine and GANT61 or AKT pathway inhibitor LY294002 were employed, and then cell proliferation and cell cycle were analyzed.

**Results:** Data from *ex vivo* culture showed that, in presence of 0.5–2.5 μM EGCG, the growth of mink hair follicles was promoted. *In vitro*, the proliferation of DPCs and ORSCs was enhanced by 0.5–4 μM EGCG treatment. More cells entered S phase upon treatment of EGCG, accompanied with upregulation of cyclin D1 and cyclin E1. Furthermore, when exposed to EGCG, the Shh and AKT signaling pathways were activated in both hair follicles and primary DPCs and ORSCs. Inhibiting either of these two pathways partly reversed the effect of EGCG on proliferation and cell cycle of DPCs and ORSCs.

**Conclusion:** EGCG promotes the growth of mink hair follicles at concentrations of 0.5–2.5 μM. This growth-promoting effect of EGCG may be associated with the increased proliferation of DPCs and ORSCs through activating Shh and AKT signaling pathways.

## Introduction

Hair follicles, which produce and moor hair shafts, are mini-organs with self-regeneration capability (Schneider et al., 2009). In postnatal life, hair follicles undergo regular cycles of anagen, catagen, and telogen. Anagen is a prolonged phase of rapid growth, with elongated and pigmented hair shafts. Then hair follicles cycle back to the relative quiescence (telogen) via an apoptosis-mediated regression (catagen) (Langan et al., 2015). Hair follicles can maintain some of the key characteristics even cultured *ex vivo* (Schneider et al., 2009). Hair follicles consist of several types of cells surrounding hair shafts, including outer root sheath cells (ORSCs), inner root sheath cells and dermal papilla cells (DPCs). Outer root sheaths, which are composed of a group of non-keratinized epithelial cells, are the connecting component between hair follicles and epidermis. ORSCs contribute to the development of hair follicles and represent a cell population with high proliferation capability during anagen phase (Vidal et al., 2005). DPCs are a group of specialized mesenchymal cells in hair follicle bulbs. In anagen, the number of DPCs is increased. They undergo asymmetric differentiation to generate progenies which are compositions of surrounding neighbors (Legue and Nicolas, 2005). DPCs also activate stem cells and lead to downward growth of follicles (Driskell et al., 2011). DPCs originating from various sources are able to induce *de novo* hair follicle formation (Jahoda et al., 1984). Thus, DPCs also play an important role in hair follicle development and contribute to hair growth (Driskell et al., 2011; Chi et al., 2013).

Epigallocatechin-3-gallate (EGCG) (**Figure 3F**) is the most abundant polyphenol in green tea, accounting for more than 50% of total polyphenols (Oliveira et al., 2016). As a major bioactive molecule in green tea, EGCG exhibits anti-inflammatory and anti-oxidative activities (Ahmed et al., 2002; Singh et al., 2002; Cavet et al., 2011; Gao et al., 2016; Shen et al., 2017), and performs protective effects in various diseases (Singh et al., 2003; Stangl et al., 2006; Wolfram et al., 2006). In addition, EGCG has been found to induce keratinocyte proliferation in human skin *in vivo* and *in vitro* (Chung et al., 2003). Importantly, Kwon et al. (2007) has reported that EGCG stimulates human hair growth via enhancing proliferation of DPCs. However, the detailed molecular mechanism of the pro-proliferative effect of EGCG has not been revealed.

The growth of hair follicles is a complex process regulated by a series of signals. The sonic hedgehog (Shh) and protein kinase B (AKT) signaling pathways are essential for the development and growth of hair follicles and transition between anagen and telogen (St-Jacques et al., 1998; Wang et al., 2000; Sohn et al., 2015). Activation of the Shh or AKT signals can enhance the proliferation of hair follicle cells and promote hair growth (Li et al., 2015; Lin et al., 2015; Sohn et al., 2015; Woo et al., 2017). EGCG has been demonstrated to trigger Shh signaling pathway in hippocampus of adult mice and enhance the hippocampal neurogenesis (Wang et al., 2012). In addition, EGCG can also activate AKT signaling pathway in various cells (Ding et al., 2017; Mi et al., 2017).

Based on these findings, we propose that these two signaling pathways may be involved in the growth-promoting effect of EGCG in hair follicles. The present study evaluated the effect of EGCG on mink hair follicles *ex vivo* and primary DPCs and ORSCs *in vitro*, and investigated the possible mechanism by focusing on the Shh and AKT signaling pathways.

## Materials and Methods

### Isolation and Culture of Hair Follicles

Anagen hair follicles were obtained from 4-month-old, male *neovison visons* (*American minks*). Small pieces of dorsal skin (∼1 cm^2^) were harvested and the hair shafts and subcutaneous fat were removed. The skin pieces were rinsed in PBS containing 100 U/ml penicillin and 0.1 mg/ml streptomycin, disinfected with iodine, discolored with 75% ethanol and then digested with Collagenase D (0.2 mg/ml; Sigma, St. Louis, MO, United States) at 4°C overnight. Subsequently, the hair follicles were isolated under a dissecting microscope and cultured in Williams E medium (Gibco, Grand Island, NY, United States) supplemented with insulin (10 μg/ml; Sigma), transferring (10 μg/ml; Sigma), hydrocortisone (10 ng/ml; Sigma), sodium selenite (10 ng/ml; Sigma), L-glutamine (2 mM; Sigma), penicillin (100 U/ml; Sigma) and streptomycin (0.1 mg/ml; Sigma) in a humid atmosphere at 37°C with 5% CO_2_ (**Supplementary Figure S8**). The hair follicles were treated with indicated concentrations of EGCG (0.1, 0.5, 1, 2.5, and 5 μM, Melonepharma, Dalian, China) for 6 days. The medium was changed every 2 days. Images of hair follicles were captured. The length of hair shafts extending from hair follicles was recorded with CellSens Standard 1.7. The daily growth of hair follicles was calculated. Daily growth of hair follicle = the length of hair follicle at one time point – the length of hair follicle at the day before. This study was performed in accordance with the Guidelines for the care and use of laboratory animals and approved by the Experimental Animal Management and Ethics Committee of the Institute of Special Animal and Plant Sciences in the Chinese Academy of Agricultural Sciences (Changchun, China).

### Isolation and Culture of DPCs and ORSCs

Dermal papilla cells were isolated according to the report of Wen et al. (2018), with some modification. Dorsal skin pieces were digested with Dispase II (0.5 mg/ml; Sigma) at 4°C overnight and incubated at 37°C for additional 30 min. Then the tissue pieces were minced and digested with Collagenase D (0.2 mg/ml) at 37°C for 6 h until the dissociation of DPCs from liquid fats can be observed under a microscope. Thereafter, the DPCs were rinsed with PBS until the supernatant was clear, filtered through a 75-μm filter and digested with trypsin. The isolated DPCs were cultured in Dulbecco’s Modified Eagle’s Medium (DMEM; Gibco) supplemented with 10% fetal bovine serum (FBS; Gibco) in a humid atmosphere at 37°C with 5% CO_2_.

Outer root sheath cells were isolated according to previously reports (Cui et al., 2012; Li et al., 2012). The hair follicles obtained as described above were seeded in a culture dish, cultured in DMEM supplemented with 10% FBS, and maintained in a humid atmosphere at 37°C with 5% CO_2_. The medium was changed every 3 days. When the ORSCs gradually attached to the dishes, the hair follicles were picked up, the ORSCs were digested with trypsin and cultured in another dish in DMEM supplemented with 10% FBS.

### Immunofluorescence

The isolated DPCs and ORSCs were characterized by α-smooth muscle actin (α-SMA) and cytokeratin 19 (CK19), respectively. Briefly, the cells were seeded onto glass slices. Then the cells were fixed in 4% paraformaldehyde for 30 min and permeabilized in 1% TritonX-100 for 30 min. Following blockade with 5% bovine serum albumin, the cells were incubated with anti-α-SMA antibody (Boster, Wuhan, China) or anti-CK19 antibody (Boster) respectively, overnight at 4°C. Then the cells were rinsed in PBS and incubated with FITC-labeled secondary antibodies (Beyotime, Haimen, China) for 60 min in the dark. Thereafter, the cells were counterstained with DAPI (Gene operation, Ann Arbor, MI, United States) and observed under a fluorescence microscope.

The isolated hair follicles were embedded with optimal cutting temperature compound (SAKURA, Torrance, CA, United States) and cut into 10 μm-sections. The sections were kept in antigen-retrieval buffer for 10 min and blocked with goat serum. Thereafter, the sections were rinsed in PBS and incubated with anti-Shh antibody (Proteintech, Wuhan, China) or anti-p-AKT antibody (Boster) overnight at 4°C. After rinsing in PBS, the sections were incubated with Cy3-labeled secondary antibodies (Beyotime) for 60 min at room temperature and counterstained with DAPI. The sections were observed under a fluorescence microscope.

### EGCG and Inhibitors Treatments

To evaluate the effect of EGCG on cell growth of DPCs and ORSCs, DPCs and ORSCs were treated with different concentrations of EGCG (0.25, 0.5, 1, 2, and 4 μM) for 12, 24, or 48 h. To investigate whether the Shh and AKT signaling pathways are involved in the effect of EGCG on growth of DPCs and ORSCs, the cells were divided into the following groups, treated accordingly and incubated for 48 h: (1) Control; (2) EGCG (0.5 μM); (3) Cyclopamine (5 μM, Medchem Express, Monmouth Junction, NJ, United States); (4) EGCG+Cyclopamine; (5) GANT61 (10 μM; Medchem Express); (6) EGCG+GANT61; (7) LY2940002 (10 μM; Beyotime); (8) EGCG+LY2940002.

### MTT Assay

Cells were seeded into 96-well plates (4 × 10^3^ cells/well). After treatments as described above, MTT (0.5 mg/ml; Sigma) was added into each well and incubated at 37°C for 4 h. Thereafter, the supernatants were removed and 200 μl dimethyl sulfoxide (Sigma) was added into each well. The optical density at 490 nm was measured with a microplate reader (BIOTEK, Winooski, VT, United States).

### Cell Cycle Detection

Cells were seeded in 6-well plates (1 × 10^5^ cells/well) and treated with EGCG or signaling pathway inhibitors as described above. Thereafter, the cells were fixed with 70% ice-cold ethanol at 4°C for 2 h. After rinsing with PBS and resuspending in 500 μl binding buffer, 25 μl propidium iodide and 10 μl RNase A were added into cells for further incubation at 37°C for 30 min in darkness. The cell cycle was then analyzed through a flow cytometer (BD, Franklin Lakes, NJ, United States).

### Quantitative Real-Time PCR (qRT-PCR)

Total RNA was extracted from hair follicles in each group using a RNA extraction kit (BioTeke, Beijing, China). The RNA was reverse transcribed into cDNA using Super M-MLV reverse transcriptase (BioTeke) and oligo(dT)_15_ according to the manufacturer’s protocol. The mRNA levels of Shh, patched (PTCH), smoothened (Smo), glioma-associated oncogene homolog 1 (Gli1) were measured by qRT-PCR (SYBR Green method) with cDNA as template and primers in **Table 1**. Relative mRNA levels were normalized to β-actin and calculated using 2^-ΔΔC_t_^ method.

**Table 1:** Sequences of primers used in quantitative real-time PCR.

Gene	Forward primer	Reverse primer
Shh	5′-TGGCTGTGGAAGC AGGTTT-3′	5′-GTCCAGGAAGGTG AGGAAGTCG-3′
PTCH	5′-GACTCCCAAGCAA ATGTATGAA-3′	5′-AGGGTCGTGGTTGT GAAGG-3′
SMO	5′-ATCGCTACCCTG CGGTTATT-3′	5′-CCAGACTACTCCAG CCATCAA-3′
Gli1	5′-CTGTCGGAAGTCCT ATTCACGC-3′	5′-CGGTCACTGGCATT GCTAAA-3′
β-actin	5′-CTGTGCCCATCTAC GAGGGCTAT-3′	5′-TTTGATGTCACGCA CGATTTCC-3′

### Western Blot

Proteins were extracted using radio immunoprecipitation assay lysis buffer with 1% phenylmethanesulfonyl fluoride (Beyotime). After measuring protein concentrations with a BCA protein assay kit (Beyotime), 30 μg proteins from each group were separated by SDS-PAGE and then transferred onto polyvinylidene fluoride membranes (Millipore, Bedford, MA, United States). Following blockade with 5% skim milk or 1% bovine serum albumin, the membranes were incubated with anti-cyclinB1, anti-cyclinD1 (1: 400; Boster), anti-PTCH (1: 500; Novus Biologicals, Littleton, CO, United States), anti-Smo (1: 100; Santa Cruz, Dallas, TX, United States), anti-Gli1 (1: 200; Santa Cruz), anti-AKT, anti-p-AKT (1: 500; Bioss, Beijing, China), anti-Shh (1: 1000; Abcam, Cambridge, United Kingdom) or anti-β-actin (1: 1000; Santa Cruz) antibodies at 4°C overnight. The membranes were rinsed in tris buffered saline with Tween (TBST) and incubated with corresponding horseradish peroxidase (HRP)-conjugated secondary antibodies (1: 5000; Beyotime) at 37°C for 45 min. Thereafter, the membranes were rinsed in TBST and visualized with an enhanced chemiluminescence detection kit (Beyotime). Relative protein levels were calculated using β-actin as the internal reference.

### Statistical Analysis

All experiments were performed three times and the results are presented as mean ± SD. Differences among groups were analyzed using One-way Analysis of Variance followed by Bonferroni’s mutiple comparison test as *post hoc*. *P* < 0.05 was considered significant.

## Results

### EGCG Promotes Hair Follicle Growth

In the present study, the effect of EGCG on hair follicle growth was investigated. The length of hair shafts extending from hair follicles was significantly increased after treatment with 0.5–2.5 μM EGCG, but a little bit of inhibition after treatment with 5 μM EGCG (**Table 2** and **Supplementary Figure S1**). The morphology of hair follicles was shown in **Supplementary Figure S9**. These results demonstrated that EGCG promoted the growth of hair follicles at a concentration of 0.5–2.5 μM.

**Table 2:** Growth of mink hair follicles.

EGCG (μM)	Daily growth (μm)						Total growth (μm)
	Day 1	Day 2	Day 3	Day 4	Day 5	Day 6	
Control	7.19 ± 2.22	6.06 ± 1.83	4.72 ± 1.09	3.94 ± 1.08	1.06 ± 0.31	1.02 ± 0.33	24.00 ± 3.76
0.1	7.50 ± 2.21	7.64 ± 1.95	3.97 ± 1.13	3.70 ± 1.03	1.17 ± 0.35	1.22 ± 0.30	25.21 ± 3.16
0.5	10.06 ± 3.13^∗^	9.69 ± 2.52^∗∗∗^	4.86 ± 1.37	3.47 ± 1.01	2.04 ± 0.63^∗∗∗^	1.13 ± 0.31	31.25 ± 5.09^∗∗∗^
1	9.36 ± 1.61	8.23 ± 2.50	3.66 ± 0.98	3.99 ± 1.21	2.73 ± 0.85^∗∗∗^	1.33 ± 0.36	29.31 ± 3.41^∗∗^
2.5	9.01 ± 2.16	7.77 ± 1.91	4.27 ± 1.32	4.34 ± 1.36	2.12 ± 0.62^∗∗∗^	1.03 ± 0.33	28.54 ± 3.67^∗^
5	4.98 ± 0.93	5.12 ± 0.88	3.02 ± 0.86^∗∗^	2.44 ± 0.71^∗∗^	1.46 ± 0.42	0.94 ± 0.27	17.97 ± 1.60^∗∗∗^

*The results are presented as mean ± SD. ^∗^p < 0.05, ^∗∗^p < 0.01, ^∗∗∗^p < 0.001 compared with the Control group*.

### EGCG Promotes the Growth of DPCs and ORSCs

**FIGURE 1:**
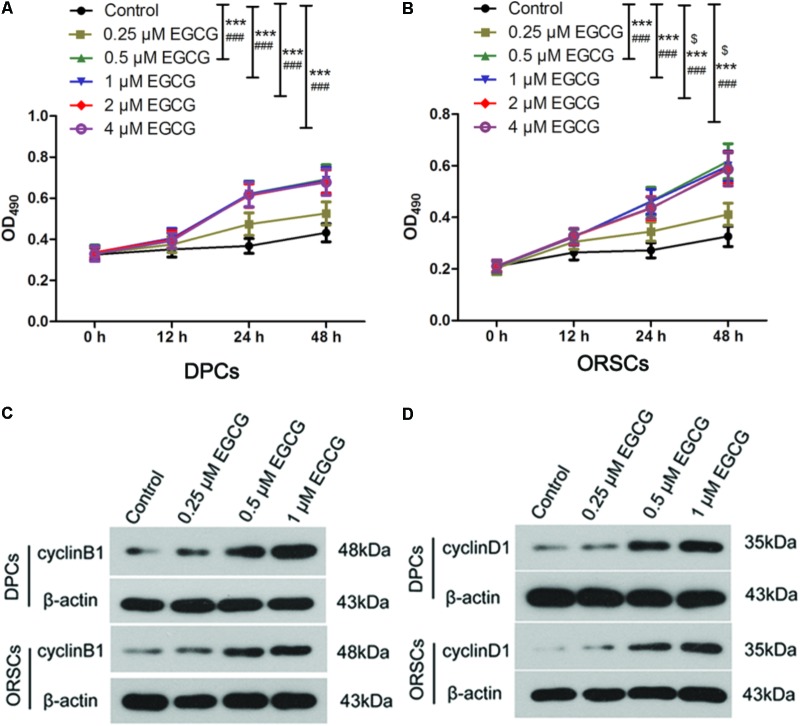
Epigallocatechin-3-gallate (EGCG) accelerates the growth of DPCs and ORSCs. **(A,B)** After treatment with 0.25, 0.5, 1, 2, and 4 μM EGCG for 12, 24, and 48 h, the cell viability of DPCs and ORSCs was assessed by MTT assay. ^$^*p* < 0.05 for 12 h, ^∗∗∗^*p* < 0.001 for 24 h, ^###^*p* < 0.001 for 48 h, all compared with the control group. **(C)** After treatment with different concentrations of EGCG, the protein level of cyclinB1 in DPCs and ORSCs was detected by western blot. β-actin served as the internal reference. **(D)** Western blot was performed to detect the protein level of cyclinD1 in DPCs and ORSCs after treatment with EGCG. All experiments were repeated three times. The results are presented as mean ± SD.

As the growth of DPCs and ORSCs is closely related to hair follicle growth, we also evaluated the effect of EGCG on the growth of DPCs and ORSCs. The isolated DPCs and ORSCs were characterized by α-SMA and CK19 respectively (**Supplementary Figure S2**). EGCG at 0.25 μM did not affect the growth of DPCs or ORSCs. While, EGCG at 0.5–4 μM significantly increased the growth of these cells compared with the control cells (**Figures 1A,B**). These results demonstrated that EGCG promoted the growth of DPCs and ORSCs at a concentration of 0.5–4 μM.

As EGCG at concentrations of 0.5, 1, 2, and 4 μM resulted in similar results, 0.25, 0.5, and 1 μM EGCG were selected for the subsequent experiments. Cell cycle is an important event that impacts cell growth. In this study, we detected the effect of EGCG on cell cycle in DPCs and ORSCs by flow cytometry. Compared with the control cells, treatment with 0.5 and 1 μM EGCG increased the proportion of cells those entered into S phase (**Table 3** and **Supplementary Figure S3A**), which indicates that EGCG accelerates the cell cycle process. Cyclins are important regulators of cell cycle. The results of western blot showed that, after treatment with 0.5 and 1 μM EGCG, the protein levels of cyclinB1 and cyclinD1 were increased significantly compared with the control cells (**Figures 1C,D** and **Supplementary Figures S3B,C**). These results provided further evidence for the growth-promoting effect of EGCG.

**Table 3:** Percentages of cells in each phase of cell cycle (%).

EGCG (μM)	DPCs			ORSCs		
	G1 phase	S phase	G2/M phase	G1 phase	S phase	G2/M phase
Control	64.07 ± 3.35	17.71 ± 1.84	17.65 ± 1.94	61.22 ± 2.67	14.03 ± 1.53	24.23 ± 3.16
0.25	52.83 ± 2.67^∗∗^	19.75 ± 1.80	26.23 ± 2.35^∗^	56.80 ± 2.09	18.73 ± 1.44	23.08 ± 3.46
0.5	47.18 ± 2.71^∗∗∗^	26.42 ± 2.42^∗∗^	25.23 ± 1.03^∗^	54.35 ± 4.20	25.71 ± 2.45^∗∗∗^	19.48 ± 2.74
1	49.52 ± 2.94^∗∗^	25.30 ± 2.65^∗^	24.10 ± 3.54	53.98 ± 3.70	25.12 ± 2.40^∗∗∗^	20.32 ± 1.31

*The results are presented as mean ± SD. ^∗^p < 0.05, ^∗∗^p < 0.01, ^∗∗∗^p < 0.001 compared with the Control group.*

### EGCG Activates the Shh and AKT Signaling Pathways

**FIGURE 2:**
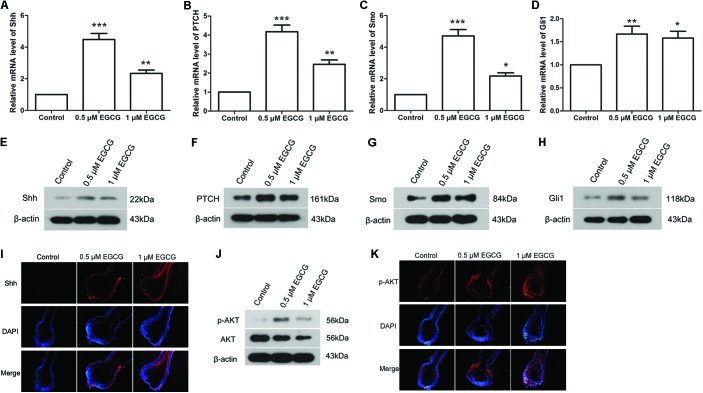
Epigallocatechin-3-gallate activates the Shh and AKT signaling pathways in hair follicles. After treatment with 0.5 and 1 μM EGCG, the mRNA levels of Shh **(A)**, PTCH **(B)**, Smo **(C),** and Gli1 **(D)** in hair follicles were detected by qRT-PCR. The relative mRNA levels were calculated using 2^-ΔΔC_t_^ method. Western blot was also performed to detect the protein levels of Shh **(E)**, PTCH **(F)**, Smo **(G),** and Gli1 **(H)** in hair follicles. β-actin served as the internal reference. **(I)** Level of Shh in hair follicles was assessed by immunofluorescence. Red fluorescence: Shh; blue fluorescence: DAPI. **(J)** The phosphorylation level of AKT was assessed by western blot. **(K)** Level of p-AKT in hair follicles was assessed by immunofluorescence. Red fluorescence: p-AKT; blue fluorescence: DAPI. Each experiment was repeated three times and the results are presented as mean ± SD. ^∗^*p* < 0.05, ^∗∗^*p* < 0.01, ^∗∗∗^*p* < 0.001 compared with the control group.

The Shh and AKT signaling pathways play important roles in hair follicle growth. In our study, the results of qRT-PCR showed that the mRNA levels of Shh, PTCH, Smo and Gli1 in hair follicles were increased to 4.48 ± 0.92-, 4.18 ± 0.85-, 4.71 ± 0.98-, and 1.67 ± 0.41-fold, respectively, after treatment with 0.5 μM EGCG, and to 2.34 ± 0.51-, 2.46 ± 0.57-, 2.18 ± 0.49-, and 1.58 ± 0.36-fold, respectively, after treatment with 1 μM EGCG (**Figures 2A–D**). The results of Western blot analysis also showed that the protein levels of Shh, PTCH, Smo and Gli1 in hair follicles were increased significantly after EGCG treatment (**Figures 2E–H** and **Supplementary Figures S4A–D**). The Shh level in hair follicles was also evaluated by immunofluorescence. As shown in **Figure 2I**, the Shh level was increased after treatment with EGCG, which was consistent with the results of western blot. These results indicated that the Shh signaling pathway was activated after treatment with EGCG. In addition, the phosphorylation level of AKT was also markedly increased to 4.91 ± 1.19-fold after treatment with 0.5 μM EGCG and to 2.30 ± 0.6-fold after treatment with 1 μM EGCG (**Figure 2J** and **Supplementary Figure S4E**). Similar results were gotten through immunofluorescence (**Figure 2K**), indicating that the AKT signaling pathway was activated.

**FIGURE 3:**
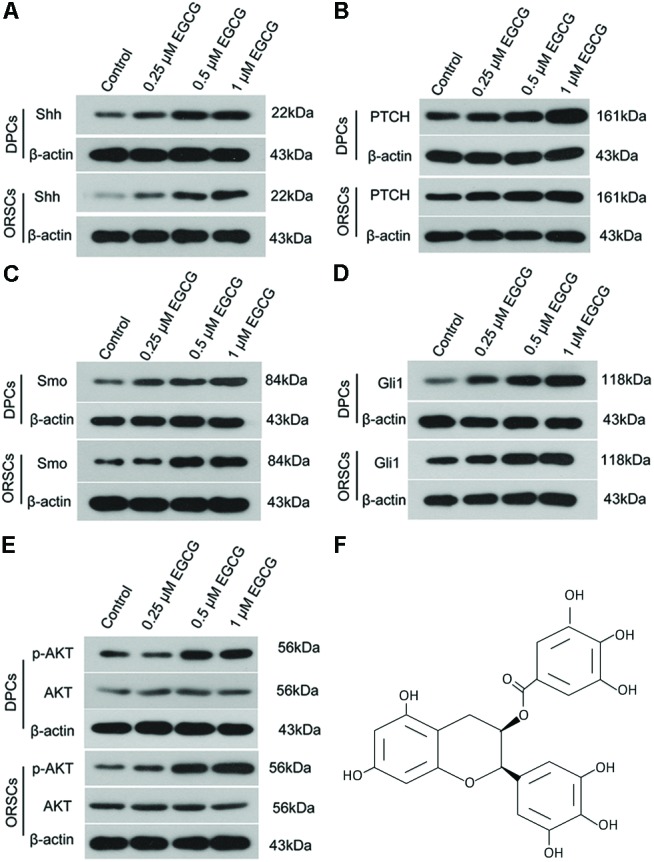
Epigallocatechin-3-gallate activates the Shh and AKT signaling pathways in DPCS and ORSCs. Upon treatment with EGCG, the protein levels of Shh **(A)**, PTCH **(B)**, Smo **(C),** and Gli1 **(D)** in DPCs and ORSCs were detected by western blot with β-actin as the internal reference. **(E)** Western blot was performed to assess the levels of AKT and p-AKT in each group with β-actin as the internal reference. **(F)** Chemical structure of EGCG. Each experiment was repeated three times.

The effect of EGCG on the Shh and AKT signaling pathways was also investigated in DPCs and ORSCs. After treatment with 0.5 μM or 1 μM EGCG, the protein levels of Shh, PTCH, Smo and Gli1 were significantly increased in both DPCs and ORSCs compared with the control cells (**Figures 3A–D** and **Supplementary Figures S5A–D**). Additionally, phosphorylated AKT level was also elevated in EGCG-treated DPCs and ORSCs (**Figure 3E** and **Supplementary Figures S5E,F**). These results suggested that the Shh and AKT signaling pathways might be involved in the effect of EGCG on hair follicle growth.

### EGCG Modulates the Growth of DPCs and ORSCs Through Shh and AKT Signaling Pathways

**FIGURE 4:**
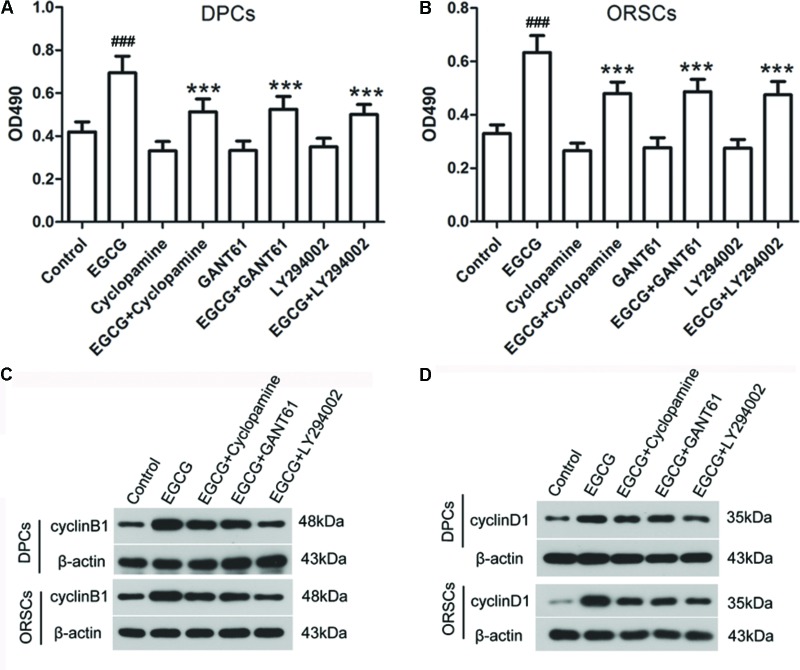
Shh and AKT signaling pathway inhibitors abolish the effect of EGCG on the growth of DPCs and ORSCs. **(A,B)** After treatment with EGCG and/or cyclopamine, GANT61 or LY294002, the cell viability of DPCs and ORSCs was assessed by MTT assay. **(C,D)** Protein levels of cyclinB1 and cyclinD1 in DPCs and ORSCs were assessed by western blot with β-actin as the internal reference. All experiments were repeated three times. The results are presented as mean ± SD. ^###^*p* < 0.001 compared with the control group; ^∗∗∗^*p* < 0.001 compared with the EGCG group.

To investigate the role of Shh and AKT signaling pathways in the growth-promoting effect of EGCG, inhibitors of Shh signaling pathway (Cyclopamine and GANT61) and AKT signaling pathway (LY2940002) were employed in this study. The effects of these inhibitors on their corresponding signaling pathways were verified (**Supplementary Figure S6**). The results of the MTT assay demonstrated that the growth-promoting effect of EGCG on DPCs and ORSCs was abolished by Cyclopamine, GANT61 or LY2940002 (**Figures 4A,B**). Meanwhile, when co-treated with Cyclopamine, GANT61 or LY2940002, the effect of EGCG on cell cycle was also blocked, as evidenced by markedly increased cell population in G0/G1 phase compared with the cells treated with EGCG alone (**Table 4** and **Supplementary Figure S7A**). Furthermore, the EGCG-induced upregulation of cyclinB1 and cyclinD1 expressions in DPCs and ORSCs were also markedly weakened by Cyclopamine, GANT61 or LY2940002 (**Figures 4C,D** and **Supplementary Figures S7B,C**). These results indicated that EGCG might modulate the growth of DPCs and ORSCs through Shh and AKT signaling pathways.

**Table 4:** Percentages of cells in each phase of cell cycle (%).

	DPCs			ORSCs		
	G1 phase	S phase	G2/M phase	G1 phase	S phase	G2/M phase
Control	65.00 ± 1.32	16.44 ± 1.12	17.85 ± 1.72	64.83 ± 2.60	13.75 ± 1.14	20.58 ± 2.05
EGCG	50.20 ± 1.83^###^	23.35 ± 2.30^##^	24.98 ± 2.40^#^	56.53 ± 3.39^#^	23.95 ± 2.85^##^	18.71 ± 1.76
Cyclopamine	69.72 ± 2.87	12.21 ± 1.52	17.65 ± 1.76	66.34 ± 2.20	10.36 ± 1.09	22.14 ± 2.46
EGCG+ Cyclopamine	64.51 ± 3.14^∗∗^	16.86 ± 1.70^∗^	18.01 ± 1.71^∗^	63.13 ± 4.05	15.28 ± 1.90^∗^	20.47 ± 2.44
GANT61	72.98 ± 2.36	12.31 ± 1.69	13.47 ± 1.94	66.10 ± 2.37	10.38 ± 1.60	22.35 ± 2.61
EGCG+ GANT61	65.66 ± 2.37^∗∗∗^	17.38 ± 2.19^∗^	16.76 ± 1.78^∗∗^	63.87 ± 1.22^∗^	14.71 ± 1.77^∗∗^	20.75 ± 2.18
LY2940002	70.24 ± 1.52	11.84 ± 1.57	17.19 ± 1.85	68.20 ± 2.87	10.21 ± 1.82	20.80 ± 2.32
EGCG+ LY2940002	56.06 ± 2.98^∗^	18.56 ± 1.91^∗^	24.50 ± 1.09	65.16 ± 3.45^∗^	15.12 ± 1.73^∗^	18.84 ± 3.77

*The results are presented as mean ± SD. ^#^p < 0.05, ^##^p < 0.01, ^###^p < 0.001 compared with the Control group. ^∗^p < 0.05, ^∗∗^p < 0.01, ^∗∗∗^p < 0.001 compared with the EGCG group*.

## Discussion

In the present study, we found that the growth of hair follicles was promoted after EGCG treatment *ex vivo*. In addition, EGCG increased the proliferation of DPCs and ORSCs, which was accompanied by the activation of Shh and AKT signals. Furthermore, inhibition of Shh and AKT signaling pathways abrogated the growth-promoting effect of EGCG. Our findings demonstrated that EGCG might promote mink hair follicle growth through Shh and AKT signaling pathways.

In our study, EGCG promoted the growth of mink hair follicles. This finding is consistent with the previous study in which EGCG promotes the growth in human hair follicles (Kwon et al., 2007). Our results confirm the growth-promoting effect of EGCG in mink hair follicles. Follicular melanogenesis, which is coupled to the anagen (Slominski et al., 2005), is also influenced by EGCG. EGCG can inhibit tyrosinase, a main enzyme in the regulation of melanin synthesis (No et al., 1999; Slominski et al., 2004), and reduce the production and secretion of melanin (Kim et al., 2004, 2018). Thus, we speculate that EGCG may also influence the melanogenesis of mink hair, but more explorations are needed.

In the present study, the growth of DPCs and ORSCs was enhanced by EGCG treatment. EGCG performs an interesting role in cell growth. EGCG is a potential anti-cancer agent, it has been demonstrated to suppress cell growth and metastasis of various cancers (Stuart et al., 2006; Gan et al., 2016; Luo et al., 2016; Shin et al., 2016; Yang et al., 2016; Zhang J. et al., 2016). In non-tumor cells, EGCG promotes the apoptosis of B lymphocytes in collagen-induced arthritis rats (Liu et al., 2012), and suppresses the antigen-induced T cell proliferation (Pae et al., 2010). Whereas, EGCG stimulates cell growth and differentiation of keratinocytes and neural stem cells (Hsu et al., 2003, 2005; Zhang Y. et al., 2016). These findings suggest that EGCG may modulate cell proliferation according to cell status. Consistent with our study, the report of Kwon et al also showed that EGCG promoted the proliferation of cultured human DPCs (Kwon et al., 2007).

Cell cycle is a vital process through which a complex multicellular organism becomes mature and injured organs are renewed. Previous studies have showed that EGCG affects cell cycle through regulating the expression of proteins associated with cell cycle, including cyclins and cyclin dependent kinases (Ahmad et al., 2002; Han et al., 2011; Lim and Cha, 2011; Zhang et al., 2012). Some reports reveal that EGCG leads to G1/S and G2/M arrest in cancer cells (Huang et al., 2009; Deng and Lin, 2011; Lim and Cha, 2011). On the contrary, our results showed that the cell cycle of DPCs and ORSCs was accelerated by EGCG, as evidenced by the increased cell population in S phase. Meanwhile, the protein levels of cyclinD1 and cyclinB1 which are closely related to G2/M and G1/S transition, were found to be increased upon the treatment of EGCG. These results demonstrated that EGCG promoted the cell cycle process of DPCs and ORSCs. This effect may be associated with the growth-promoting effect of EGCG on hair follicles of mink.

The Shh signaling pathway plays a critical role in vertebrate organogenesis and earlier studies show that it also plays an important role in hair growth (Gritli-Linde et al., 2007). Lack of Shh or Smo has been reported to impair the growth, morphogenesis and differentiation of hair follicles (St-Jacques et al., 1998; Chiang et al., 1999; Gritli-Linde et al., 2007). The Shh signaling cascade is initiated by the binding of functional Shh ligand to its receptor PTCH. The binding activates Smo by dissociating it from inhibition by PTCH and triggers the downstream transcription factors Gli. In our study, the levels of key proteins in the Shh signaling pathway, including Shh, PTCH, Smo and Gli1 were significantly upregulated upon EGCG treatment, both in hair follicles and in primary DPCs and ORSCs, indicating that the Shh signaling pathway was activated by EGCG. This observation is consistent with the previous finding in hippocampal neural progenitor cells (Wang et al., 2012). In addition, the Shh signaling pathway also has closely association with the cell cycle checkpoints. First, PTCH participates in the G2/M checkpoint through regulating cyclin B1 nuclear translocation. The binding of Shh ligand with PTCH facilitates cyclin B1 to localize to the nucleus (Barnes et al., 2005). Second, activated Gli directly upregulates cyclinD1 expression and enhances cyclinB1 expression via FOXM1 (Kasper et al., 2006; Katoh and Katoh, 2009). Here we found that EGCG enhanced the expressions of cyclinB1 and cyclinD1, and Shh inhibitors abolished this enhancement and blocked the growth-promoting effect of EGCG. These findings suggest that the activation of Shh signaling pathway may contribute to the growth-promoting effect of EGCG on mink hair follicles through regulating cell cycle checkpoint proteins. AKT, a downstream effector of phosphatidylinositol 3-kinase, is implicated in growth and survival of a variety of cells (Hers et al., 2011). Activation of AKT appears to contribute to hair growth (Sohn et al., 2015; Woo et al., 2017). Numerous studies have reported that EGCG regulates AKT signaling pathway. In line with these studies, we found that the AKT signaling pathway is activated by EGCG in cultured mink hair follicles and primary DPCs and ORSCs. In addition, AKT also regulates the expression of cyclins through glycogen synthase kinase-3β (Li et al., 2017), which is consistent with our observations that AKT signaling pathway inhibitor abated the growth-promoting effect of EGCG on hair follicles and cells, and downregulated cyclin expressions. These results suggest that the activation of the AKT signaling pathway may be involved in the effect of EGCG on mink hair follicle growth.

We realized that Shh and AKT signaling pathways might be not the only signaling pathways underlying the effect of EGCG. Other signaling pathways, such as Wnt, Notch and bone morphogenetic protein, are implicated in the development of hair follicles (Rishikaysh et al., 2014) and influenced by EGCG in several other reports (Jin et al., 2013; Fujita et al., 2017; Zhu et al., 2017). Thus, these signaling pathways may be also involved in the growth-promoting effect of EGCG on mink hair follicle growth, but more explorations are needed.

## Conclusion

Epigallocatechin-3-gallate promoted the growth of mink hair follicles *in vitro*, and increased the proliferation of DPCs and ORSCs through activating the Shh and AKT signaling pathways.

## Author Contributions

HZ and GL designed and conducted the study, analyzed the data, and prepared the manuscript. WN, SW, XS, HS, and TL collected and interpreted the data.

## Conflict of Interest Statement

The authors declare that the research was conducted in the absence of any commercial or financial relationships that could be construed as a potential conflict of interest.

## Supplementary Material

FIGURE S1 Epigallocatechin-3-gallate (EGCG) promotes the growth of hair follicles. Hair follicles were treated with 0.1, 0.5, 1, 2.5, or 5 μM of EGCG, and then images of hair follicles were captured on Day 0, Day 2, Day 4, and Day 6.

FIGURE S2 Characteristics of DPCs and ORSCs. **(A)** The isolated DPCs were characterized by α-SMA through immunofluorescence. Green fluorescence: α-SMA. Scale bar = 200 μm. **(B)** Identities of ORSCs. Green fluorescence: CK19. Scale bar = 100 μm.

FIGURE S3 Epigallocatechin-3-gallate accelerates the cell cycle of DPCs and ORSCs. **(A)** After treatment with 0.25, 0.5, and 1 μM EGCG for 48 h, the cell cycle distribution was detected by flow cytometry. **(B,C)** Western blot was performed to detect the protein levels of cyclinB1 and cyclinD1 in DPCs and ORSCs after treatment with EGCG. The relative protein levels of cyclinB1 and cyclinD1 were calculated according to the results of western blot. ^∗∗∗^*p* < 0.001 compared with the control group. The results are presented as mean ± SD.

FIGURE S4 Epigallocatechin-3-gallate activates the Shh and AKT signaling pathways in hair follicles. Western blot was performed to detect the protein levels of Shh **(A)**, PTCH **(B)**, Smo **(C),** and Gli1 **(D)**, and phosphorylation level of AKT **(E)** in hair follicles. The relative levels were calculated according to the results of western blot. The results are presented as mean ± SD. ^∗^*p* < 0.05, ^∗∗∗^*p* < 0.001 compared with the control group.

FIGURE S5 Epigallocatechin-3-gallate activates the Shh and AKT signaling pathways in DPCS and ORSCs. Upon treatment with EGCG, the protein levels of Shh **(A)**, PTCH **(B)**, Smo **(C),** and Gli1 **(D)** in DPCs and ORSCs were detected by western blot. **(E, F)** Western blot was performed to assess the levels of AKT and p-AKT in each group. Relative levels were calculated according to the results of western blot. The results are presented as mean ± SD. ^∗^*p* < 0.05, ^∗∗^*p* < 0.01, ^∗∗∗^*p* < 0.001 compared with the control group.

FIGURE S6 The effects of inhibitors Cyclopamine, GANT61 and LY2940002. **(A,B)** Cyclopamine and GANT61 decreased the level of Gli1 as evidenced by western blot. β-actin served as the internal reference. **(C,D)** LY2940002 decreased the phosphorylation of AKT. Western blot was carried out to detect the phosphorylation level of AKT. β-actin served as the internal reference. All experiments were repeated three times. The results are presented as mean ± SD. ^∗∗∗^*p* < 0.001 compared with the control group.

FIGURE S7 Shh and AKT signaling pathway inhibitors abolish the effect of EGCG on the growth of DPCs and ORSCs. **(A)** Cell cycle distribution in DPCs and ORSCs in each group was detected by flow cytometry. **(B,C)** Protein levels of cyclinB1 and cyclinD1 in DPCs and ORSCs were assessed by western blot. Relative protein levels of cyclinB1 and cyclinD1 were calculated according to the results of western blot. The results are presented as mean ± SD. ^###^*p* < 0.001 compared with the control group; ^∗^*p* < 0.05, ^∗∗^*p* < 0.01, ^∗∗∗^*p* < 0.001 compared with the EGCG group.

FIGURE S8 Isolation of mink hair follicles.

FIGURE S9 HE staining for hair follicles upon treatment with EGCG.
