# Supplementary figures and images for: Epigallocatechin-3-Gallate Promotes the Growth of Mink Hair Follicles Through Sonic Hedgehog and Protein Kinase B Signaling Pathways
Source: Front Pharmacol. 2018 Jun 26;9:674. doi: 10.3389/fphar.2018.00674 (PMC6028712; doi:10.3389/fphar.2018.00674)

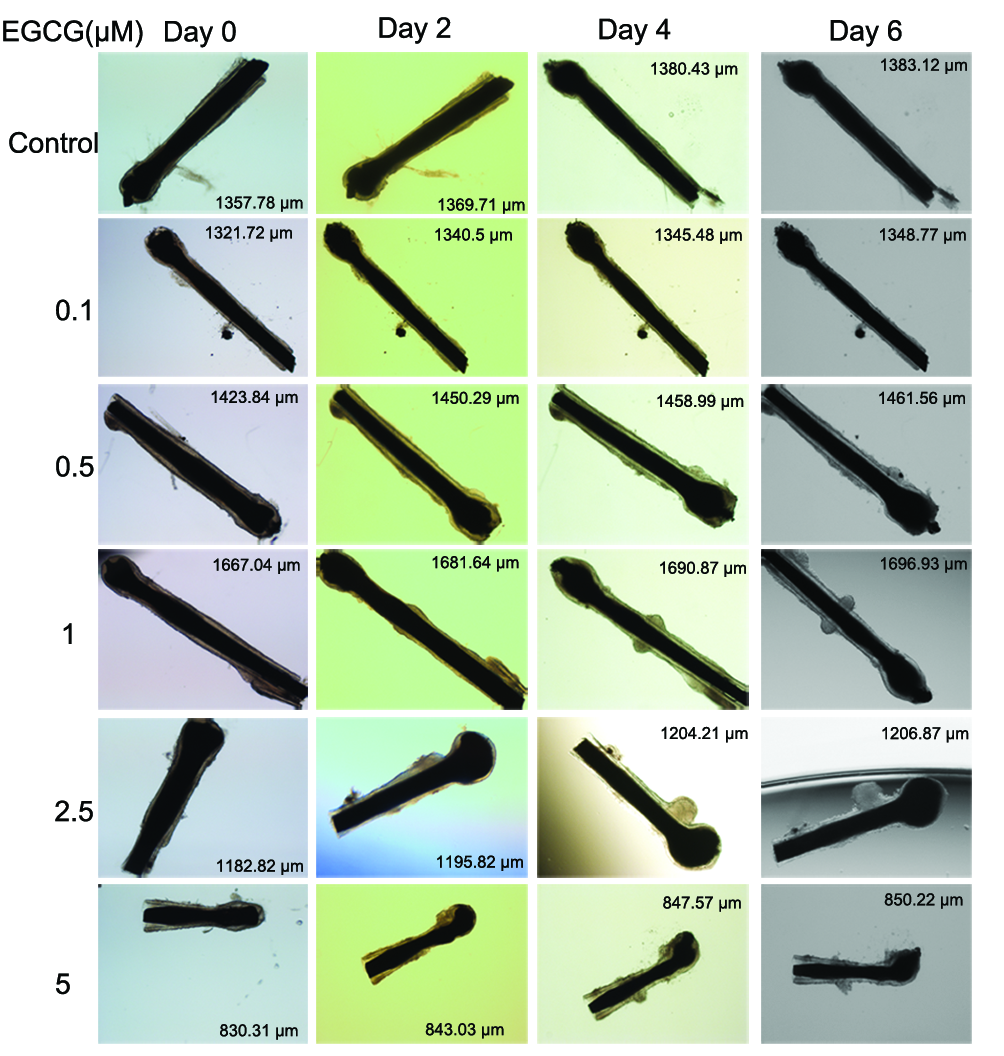

Supplement: FIGURE S1 — Epigallocatechin-3-gallate (EGCG) promotes the growth of hair follicles. Hair follicles were treated with 0.1, 0.5, 1, 2.5, or 5 μM of EGCG, and then images of hair follicles were captured on Day 0, Day 2, Day 4, and Day 6. [file Image_1.TIF]

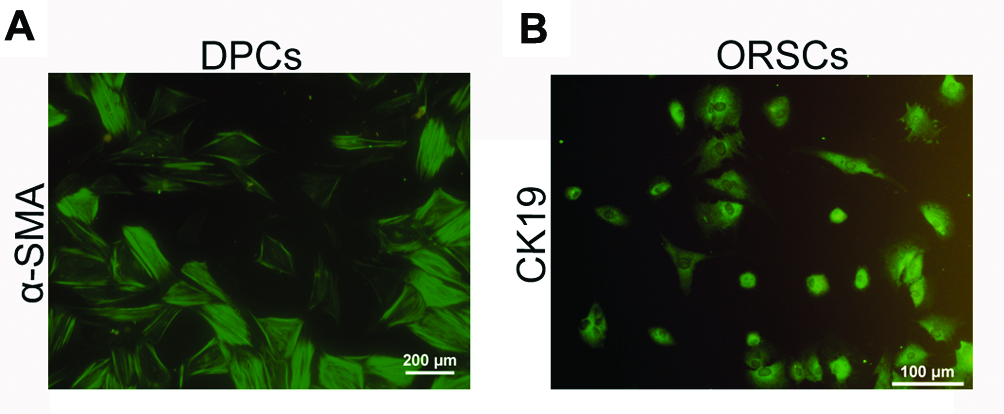

Supplement: FIGURE S2 — Characteristics of DPCs and ORSCs. (A) The isolated DPCs were characterized by α-SMA through immunofluorescence. Green fluorescence: α-SMA. Scale bar = 200 μm. (B) Identities of ORSCs. Green fluorescence: CK19. Scale bar = 100 μm. [file Image_2.TIF]

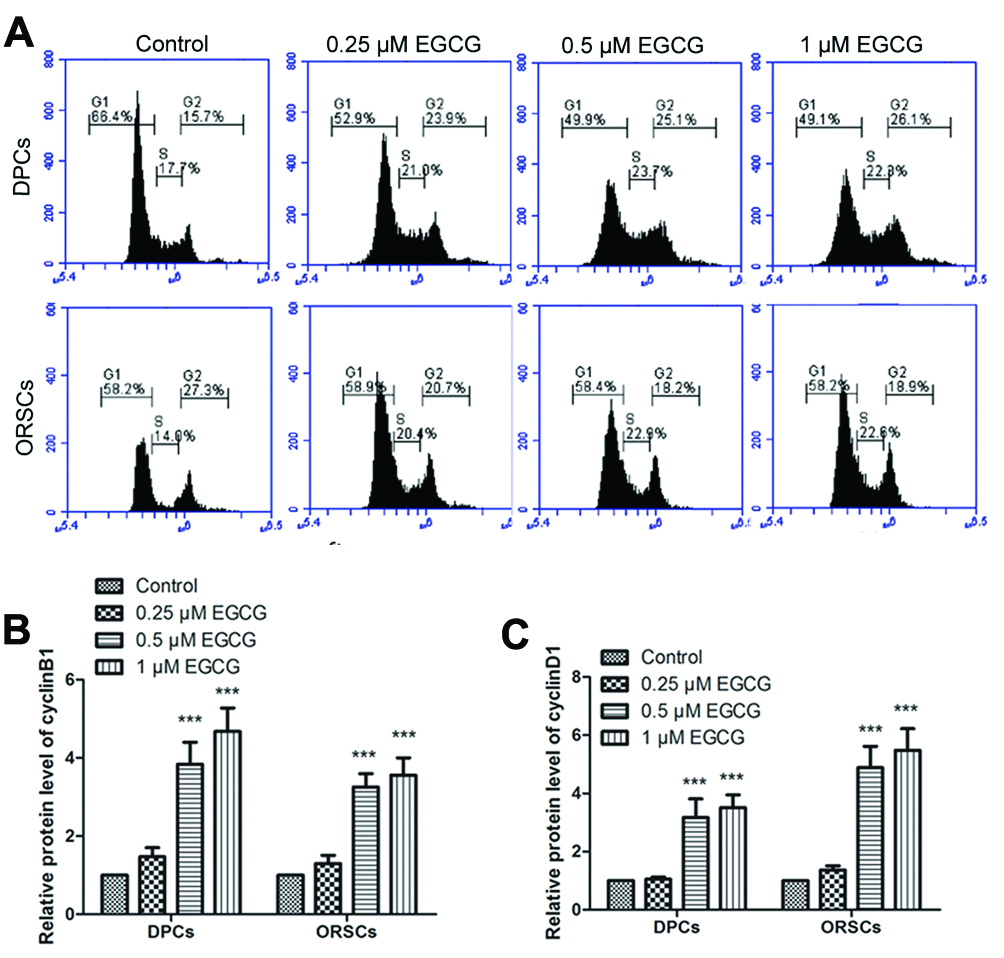

Supplement: FIGURE S3 — Epigallocatechin-3-gallate accelerates the cell cycle of DPCs and ORSCs. (A) After treatment with 0.25, 0.5, and 1 μM EGCG for 48 h, the cell cycle distribution was detected by flow cytometry. (B,C) Western blot was performed to detect the protein levels of cyclinB1 and cyclinD1 in DPCs and ORSCs after treatment with EGCG. The relative protein levels of cyclinB1 and cyclinD1 were calculated according to the results of western blot. ∗∗∗p < 0.001 compared with the control group. The results are presented as mean ± SD. [file Image_3.TIF]

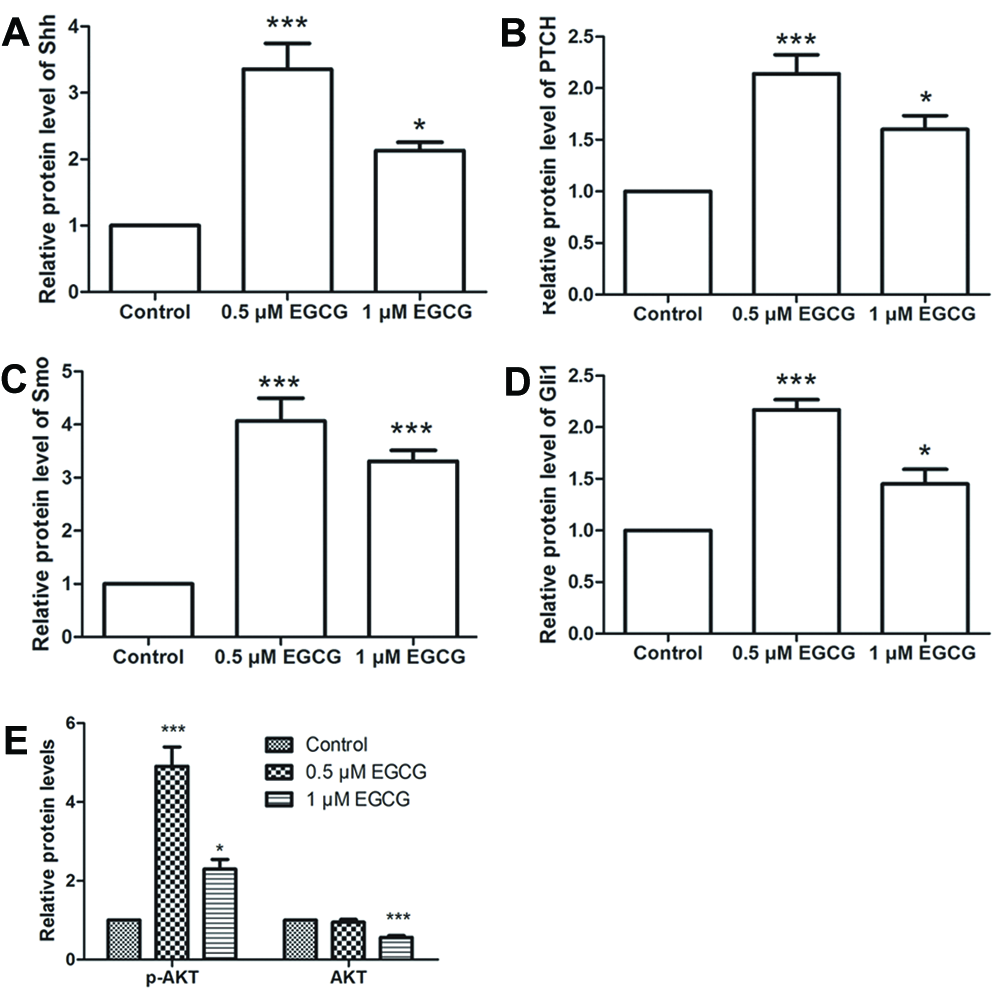

Supplement: FIGURE S4 — Epigallocatechin-3-gallate activates the Shh and AKT signaling pathways in hair follicles. Western blot was performed to detect the protein levels of Shh (A), PTCH (B), Smo (C), and Gli1 (D), and phosphorylation level of AKT (E) in hair follicles. The relative levels were calculated according to the results of western blot. The results are presented as mean ± SD. ∗p < 0.05, ∗∗∗p < 0.001 compared with the control group. [file Image_4.TIF]

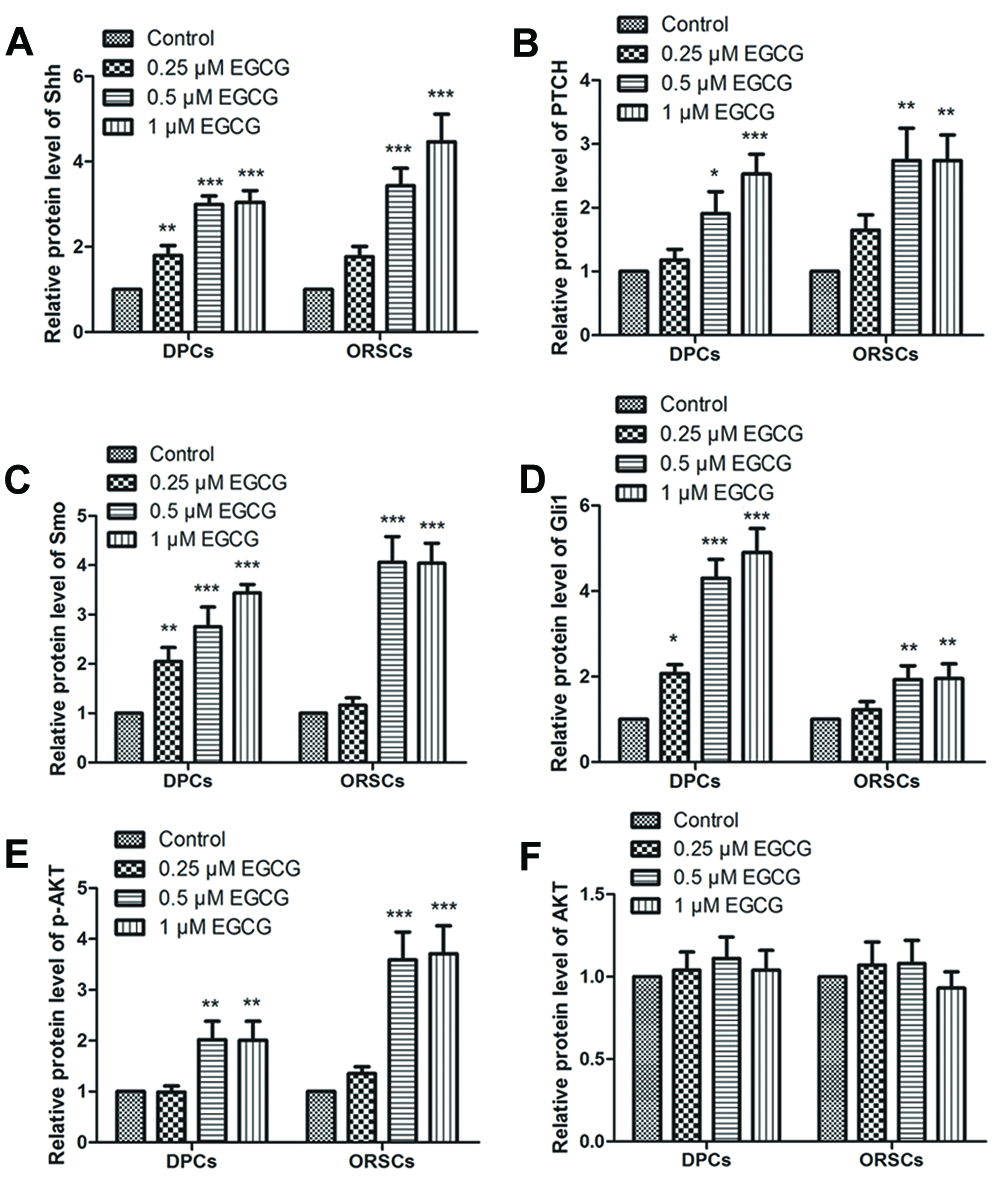

Supplement: FIGURE S5 — Epigallocatechin-3-gallate activates the Shh and AKT signaling pathways in DPCS and ORSCs. Upon treatment with EGCG, the protein levels of Shh (A), PTCH (B), Smo (C), and Gli1 (D) in DPCs and ORSCs were detected by western blot. (E, F) Western blot was performed to assess the levels of AKT and p-AKT in each group. Relative levels were calculated according to the results of western blot. The results are presented as mean ± SD. ∗p < 0.05, ∗∗p < 0.01, ∗∗∗p < 0.001 compared with the control group. [file Image_5.TIF]

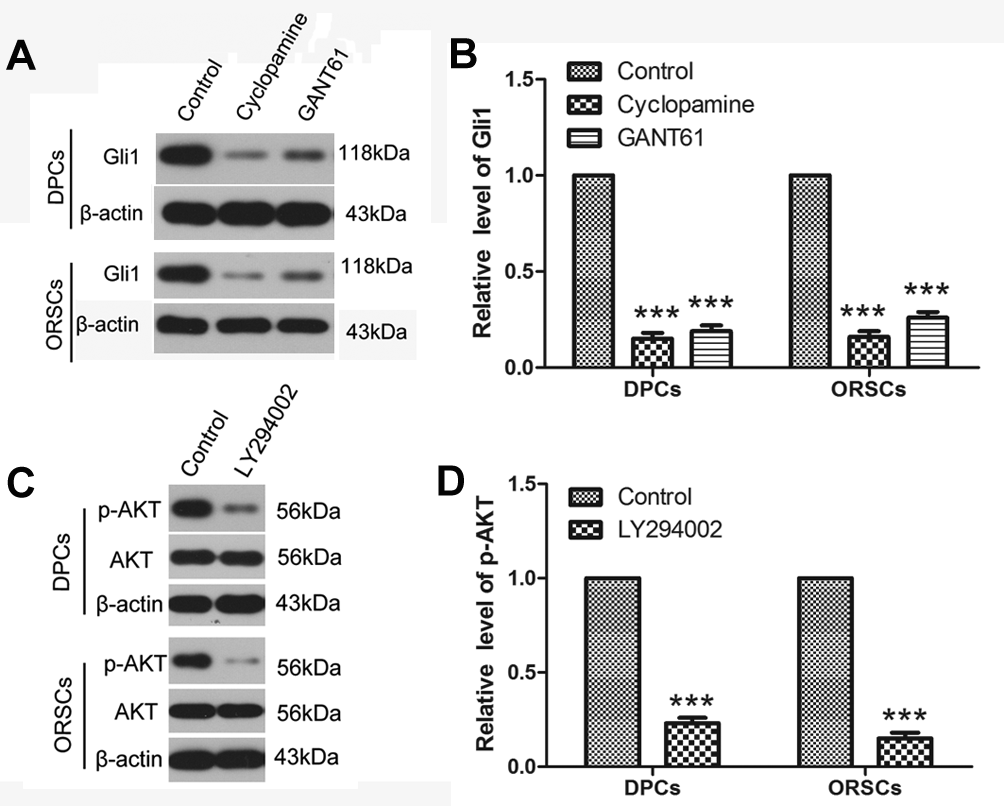

Supplement: FIGURE S6 — The effects of inhibitors Cyclopamine, GANT61 and LY2940002. (A,B) Cyclopamine and GANT61 decreased the level of Gli1 as evidenced by western blot. β-actin served as the internal reference. (C,D) LY2940002 decreased the phosphorylation of AKT. Western blot was carried out to detect the phosphorylation level of AKT. β-actin served as the internal reference. All experiments were repeated three times. The results are presented as mean ± SD. ∗∗∗p < 0.001 compared with the control group. [file Image_6.TIF]

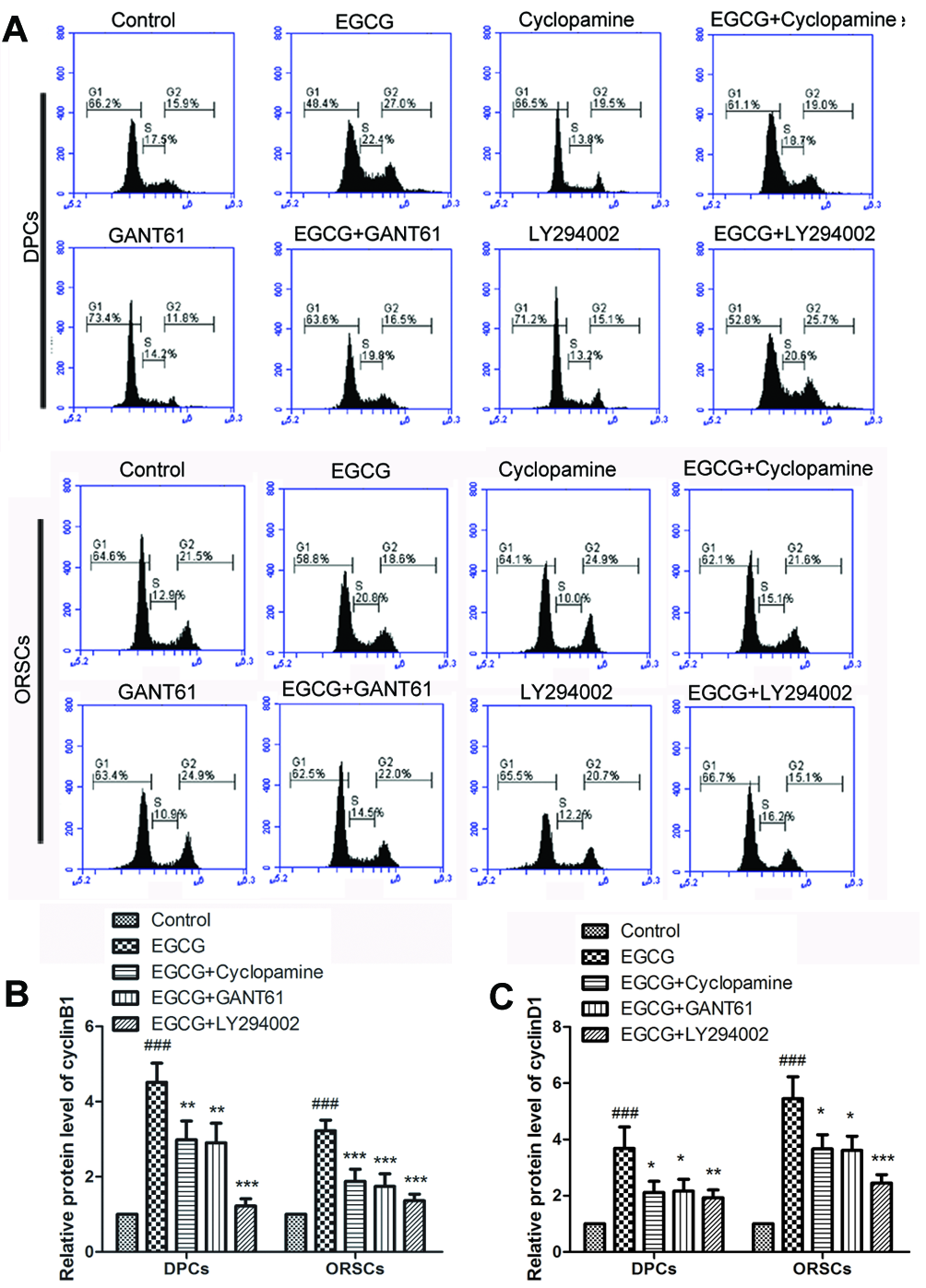

Supplement: FIGURE S7 — Shh and AKT signaling pathway inhibitors abolish the effect of EGCG on the growth of DPCs and ORSCs. (A) Cell cycle distribution in DPCs and ORSCs in each group was detected by flow cytometry. (B,C) Protein levels of cyclinB1 and cyclinD1 in DPCs and ORSCs were assessed by western blot. Relative protein levels of cyclinB1 and cyclinD1 were calculated according to the results of western blot. The results are presented as mean ± SD. ###p < 0.001 compared with the control group; ∗p < 0.05, ∗∗p < 0.01, ∗∗∗p < 0.001 compared with the EGCG group. [file Image_7.TIF]

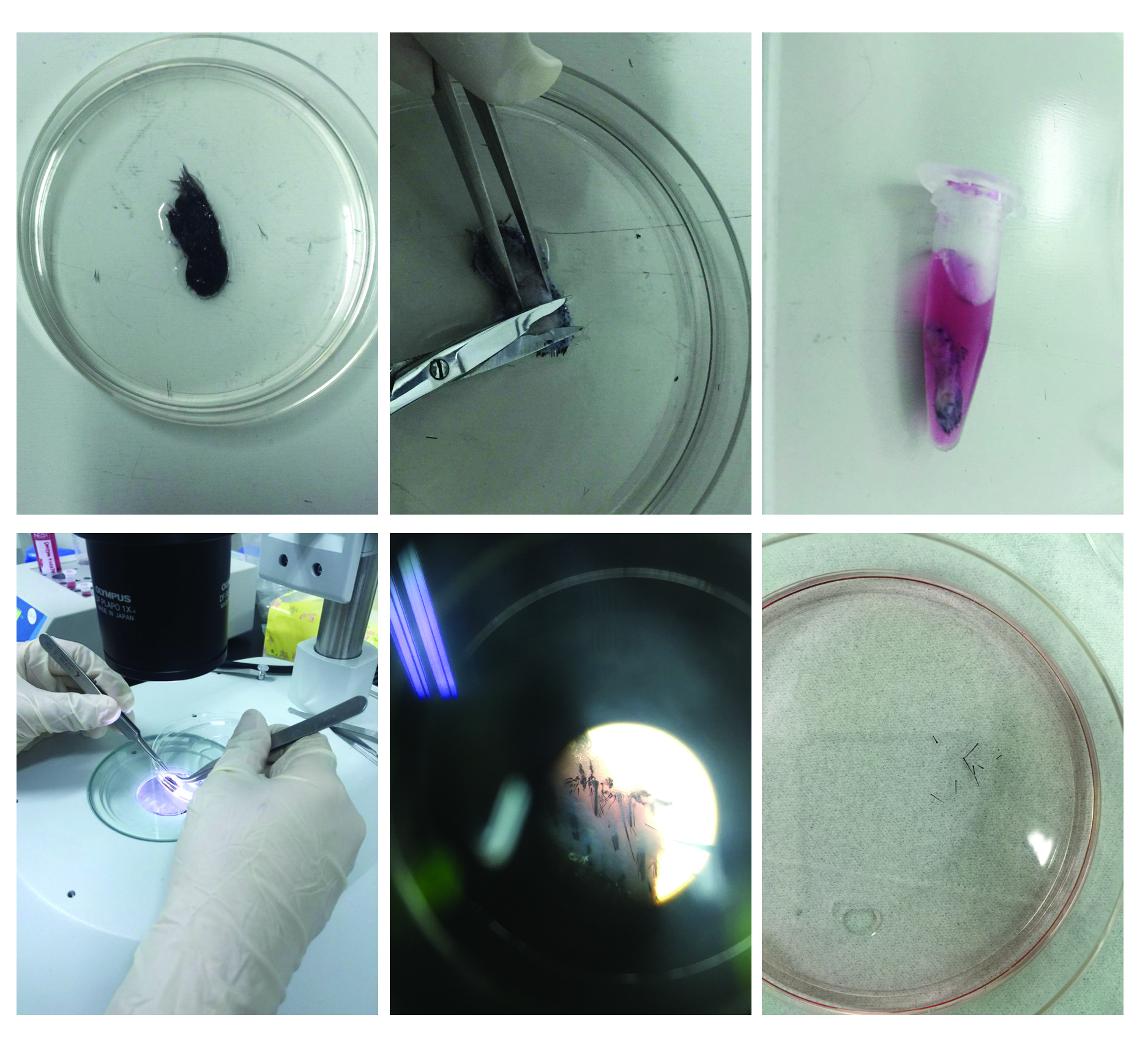

Supplement: FIGURE S8 — Isolation of mink hair follicles. [file Image_8.TIF]

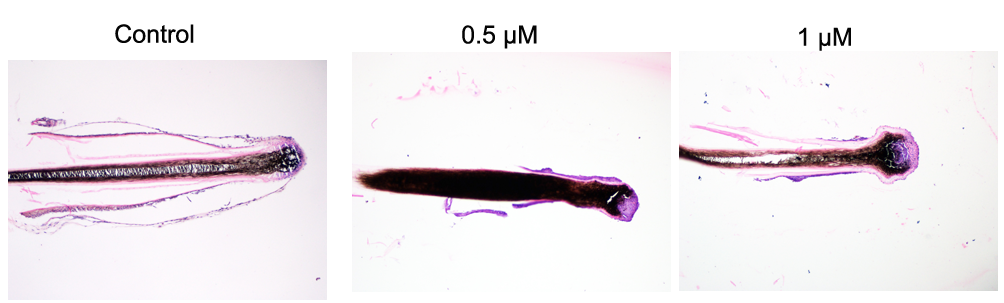

Supplement: FIGURE S9 — HE staining for hair follicles upon treatment with EGCG. [file Image_9.TIF]
